# Granular cell tumors of the urinary bladder

**DOI:** 10.1186/1477-7819-5-33

**Published:** 2007-03-13

**Authors:** Farhat Abbas, Amanullah Memon, Tanya Siddiqui, Naila Kayani, Nazim Ali Ahmad

**Affiliations:** 1 Department of Surgery (Urology), The Aga Khan University Hospital, Karachi, Pakistan; 2 Department of Pathology, The Aga Khan University Hospital, Karachi, Pakistan

## Abstract

**Background:**

Granular cell tumors (GCTs) are extremely rare lesions of the urinary bladder with only nine cases being reported in world literature of which one was malignant. Generally believed to be of neural origin based on histochemical, immunohistochemical, and ultrastructural studies; they mostly follow a clinically benign course but are commonly mistaken for malignant tumors since they are solid looking, ulcerated tumors with ill-defined margins.

**Materials and methods:**

We herein report two cases of GCTs, one benign and one malignant, presenting with gross hematuria in a 14- and a 47-year-old female, respectively.

**Results:**

Histopathology revealed characteristic GCTs with positive immunostaining for neural marker (S-100) and negative immunostaining for epithelial (cytokeratin, Cam 5.2, AE/A13), neuroendocrine (neuron specific enolase, chromogranin A, and synaptophysin) and sarcoma (desmin, vimentin) markers. The benign tumor was successfully managed conservatively with transurethral resection alone while for the malignant tumor, radical cystectomy, hysterectomy with bilateral salpingo-oophorectomy, anterior vaginectomy, plus lymph node dissection was done. Both cases show long-term disease free survival.

**Conclusion:**

We recommend careful pathologic assessment for establishing the appropriate diagnosis and either a conservative or aggressive surgical treatment for benign or localized malignant GCT of the urinary bladder, respectively.

## Background

Granular cell tumors are unusual, rare neoplasm that most commonly affect the head and neck region, especially the tongue. Only nine cases of granular cell tumors of the urinary bladder have thus far been reported. Although usually benign, these lesions may present as solid tumors with ill-defined margins and ulcerated surface, masquerading as a malignant tumor at initial presentation, and hence could be confused with transitional cell/squamous cell carcinoma or sarcoma in the urinary bladder. Only one case of malignant granular cell tumor of the urinary bladder has thus far been reported. We herein, report two cases of granular cell tumor of the urinary bladder, one benign and one malignant, and review the literature with a view to comment on the existing experience about the presentation, diagnosis and management of this rare bladder tumor.

## Case presentations

### Case-1

A 14-year-old girl presented to the Emergency Room in December 1999 with her first episode of severe gross, painless hematuria. Past history was unremarkable. On examination she was pale, dehydrated and continually bleeding *per urethram*. Heart rate was 120/minute, blood pressure 90/55 mmHg and she was afebrile. Hemoglobin was 5.9 gm/dl, hematocrit 18 with coagulation profile and routine chemistry being normal. Urinalysis showed large amounts of RBC's and 08 WBC's/HPF. Urine culture and sensitivity was later negative for bacterial growth. At emergency cystoscopy following resuscitation, she was found to have an 8 × 10 cm highly vascular, solid, infiltrative tumor occupying the right lateral wall and ipsilateral half of the trigone. The tumor involved the right ureteric orifice and extended to 1 cm short of the bladder neck. Preoperative intravenous pyelogram showed a non-excretory right kidney due to hydronephrosis with normal contralateral system. Transurethral resection of the tumor till bladder wall level was done. She needed 5 units of packed cells transfusion. Subsequently, right percutaneous nephrostomy was done, which was later internalized with a double J stent.

#### Pathologic findings

**Figure 1:**
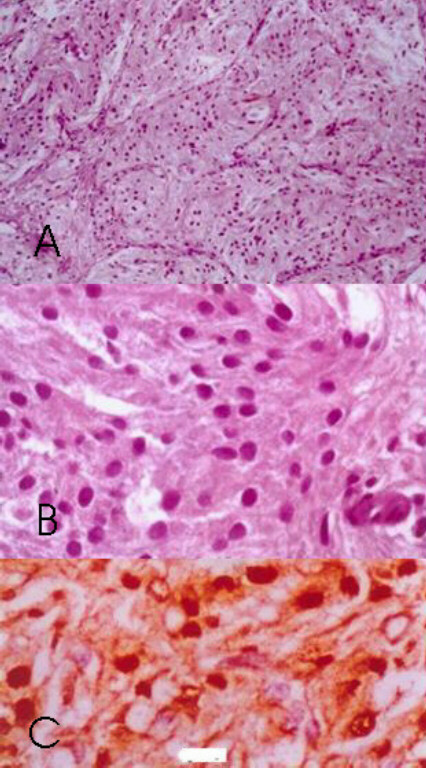
**(a): **Histology of benign granular cell tumor of the bladder of patient 1 (haematoxylin and eosin stain ×100); **b): **Histology of benign granular cell tumor of the bladder of patient1 (haematoxylin and eosin stain ×100); **c): **Immunohistochemical staining of benign granular cell tumor of the bladder of patient 1. (S-100 stain) Note positive staining of cell nucleus and cytoplasm. (×100).

Microscopic examination of the tissue revealed cohesive groups of cells arranged in lobules by dividing fibrous septae. There was no evidence of muscle invasion. The cells contained abundant granular eosinophilic cytoplasm with round monomorphic nuclei and showed focal positivity with PAS stain (Figure 1a &1b). Immunohistochemical studies revealed diffuse positivity with S-100 protein, and negativity with Desmin and Vimentin, thus consistent with granular cell tumor instead of the suspected sarcoma. Positivity with neuron specific enolase, chromogranin and synaptophysin was not seen and S-100 staining was seen in tumor cells and not in sustentacular cells as noted in pheochromocytoma

#### Course

**Figure 2:**
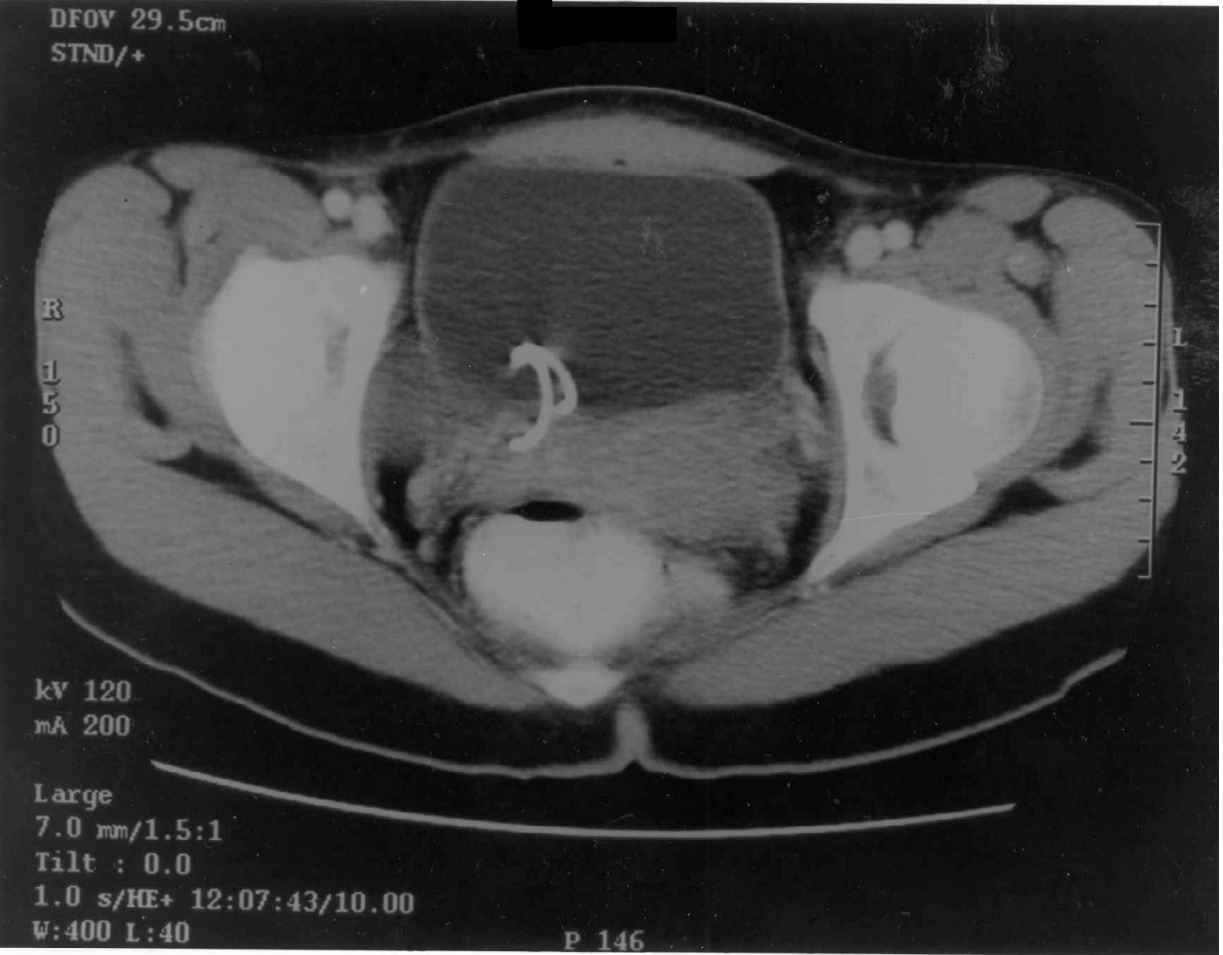
CT scan of the abdomen/pelvis at 4 months post op showing a normal-looking bladder with right double J stent in place.

CT scan of the abdomen/pelvis and chest X-ray showed no metastases. There was localized thickening of the bladder wall with no extravesical extension. At repeat cystoscopy 2 weeks later, there was no obvious residual tumor and deep biopsies from previous resection site were clear. Hence, it was decided to treat the patient conservatively. Follow-up CT scan of the abdomen/pelvis 4 months later showed a normal-looking bladder (Figure 2). Cystoscopy with multiple bladder biopsies was again negative for tumor recurrence and only showed acute on chronic inflammation. The double J stent was removed. Follow up IVP at 12 months was normal. She now remains free of disease at over 4 years since the operation.

### Case-2

A 47-year-old lady, married with 6 children, diabetic and hypertensive, presented to the urology clinic in July 1995 with gross intermittent hematuria and dysuria for one year. She also complained of urgency and urge incontinence. About 25 years back, she had a laparotomy for intestinal obstruction secondary to tuberculosis and had received a one year course of antituberculous medical therapy. Physical examination was unremarkable except for some fullness in the left lower quadrant of abdomen. Hemoglobin was 8.5 gm/dl, hematocrit 26.2 (normal = 35.4 – 42.8%) with coagulation profile and routine chemistry being normal. Urinanalysis showed hematuria and pyuria while urine culture was negative for bacterial growth. Ultrasound revealed a 2.6 × 1.8 cm polypoidal mass in the left posterior wall of urinary bladder with bladder wall irregularity. An IVP showed normal upper urinary tracts with a filling defect in the bladder.

Cystoscopy revealed an approximately 4 × 3 × 3 cm solid tumor in the left postero-lateral wall, above the ureteric orifice. Multiple biopsies of the tumor from the base and edges, as well as random bladder biopsies, were obtained.

#### Pathologic findings

**Figure 3:**
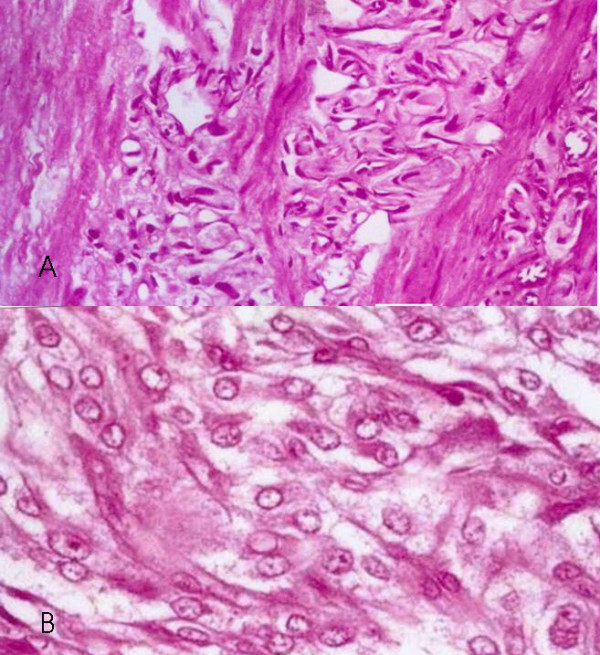
**(a): **Histology of malignant granular cell tumor of the bladder of patient 2 (haematoxylin and eosin stain). Note infiltration of tumor cells into the smooth muscle. (×100); **b): **Histology of malignant granular cell tumor of the bladder of patient 2 (haematoxylin and eosin stain). The cells exhibit abundant pink granular cytoplasm. Note prominent nucleoli in majority of cells. (×100);

The tumor was arranged in large clusters with diffuse sheets invading and insinuating in between the smooth muscle fibers. The tumor cells exhibited pleomorphism, nuclear hyperchromasia, with 2–3 mitoses/10 HPF (Figure 3a &3b). Random bladder biopsies were normal. Immunohistochemical studies showed the tumor cells to be S-100 positive and cytokeratin Cam 5.2, AE/A13 negative, illustrating a non-epithelial origin. The pathologic diagnosis of a muscle-invasive, malignant granular cell tumor of the urinary bladder was made. Neuroendocrine markers such as neuron specific enolase, chromogranin, synaptophysin were negative.

#### Course

A radionuclide bone scan was negative for metastasis. A CT scan of abdomen and pelvis showed no extravesical tumor extension or distant metastasis. A cystic left ovarian mass was recognized, which later turned out to be benign ovarian cyst with no malignancy. A planned radical cystectomy, bilateral salpingo-oophorectomy, hysterectomy, ileal conduit and pelvic lymph node clearance with resection of the anterior wall of the vagina was carried out in June 1995 realizing the malignant nature of the tumor. She made an uneventful recovery and histopathology confirmed a pT3N0M0 malignant granular cell tumor of the urinary bladder. She remains free of disease recurrence at 8 years since the operation.

## Discussion

Granular cell tumor (GCT) is an extremely rare neoplasm affecting the urinary bladder with only 9 cases described so far (Table 1) [1-9]. Although the large majority follows a clinically benign course, they are commonly mistaken for malignant tumors at the outset, as they are usually solid looking, ulcerated tumors with ill-defined margins.

**Table 1:** Summary of Granular cell tumor of the urinary bladder cases

**References**	**Age/Sex**	**Histologic Diagnosis**	**Surgery**	**Recurrence**	**Follow up**
Ravich *et al*, 1945[1]	31 y/M	MGCT	Complete Excision	Yes	Died from recurrence + metastases at 17 months
Anderson and Hoeg, 1968[2]	61 y/M	BGCT	Complete Trans-urethral resection	No	Well for 2 years
Seery, 1968[3]	31 y/F	BGCT	Segmental resection	No	Well for 18 years
Okuda *et al*, 1969[4]	49 y/F	BGCT	Partial cystectomy	No	Well for 1 year
Christ and Ozzello, 1971[5]	23 y/F	BGCT	Complete Resection	(not stated)	(course unknown)
Mouradian *et al*, 1974[6]	26 y/F	BGCT	Complete Trans-urethral resection.	Yes: at 10 and 17 months post-op.	Disease free after recurrences for 2.5 years
Fletcher *et al*, 1985[7]	48 y/M	BGCT	Trans-urethral resection + suprapubic exploration and drainage of perivesical space.	No	(time period not stated)
Kontani *et al*, 1999[8]	59 y/F	BGCT	Complete TURBT	Yes: at 7 months post-op.	Disease free for 18 months after 2nd surgery
Yoshida *et al*, 2001[9]	48/M	BGCT	TURBT × 2	Yes: average 6 months. No recurrence for 3 yrs after 2nd TURBIT	Well for 3 1/2 yrs.
Present series	14/F	BGCT	Complete TURBT	No	NED for 4 years.
Present series	47/F	MGCT	Radical cystectomy, hysterectomy, bilateral salpingo-oophorectomy + pelvic node dissection.	No	NED for 4 years.

*MGCT = Malignant granular cell tumor

** BGCT = Benign granular cell tumor

The entity was originally described by Abrikosoff [10] in 1926 as originating from myoblasts, granular cell tumors are now generally believed to have a neural (probably the Schwann cell) precursor based on histochemical, immunohistochemical and ultrastructural findings [6,11]. However, in some lesions there is no evidence of Schwann cell proliferation [12]. They occur more commonly at other sites with 30–50% of cases reported in the head and neck region [13]. The lesions can be multiple and multifocal. Middle-aged males are most commonly affected though these tumors can occur in persons of all ages. Depending on the site and size of these tumors, the patients can present from asymptomatic nodules to symptoms due to pressure effects on vital structures such as the trachea, esophagus and pituitary gland. Gross hematuria is the most common symptom in patients affected with granular cell tumor of the urinary bladder.

Histologically, Granular cell tumor of the urinary bladder is indistinguishable from similar tumors occurring at other sites. Immunohistochemical studies are particularly useful to differentiate such tumors from carcinomas and sarcomas as GCTs reveal positive staining for S-100 protein, calretinin, alpha subunit of inhibin HLA-DR, laminin and various myelin proteins [14]. The cells do not react with antibodies for neurofilaments proteins or glial fibrillary acidic protein [15,16]. Non reactivity to epithelial and muscle markers differentiates them from carcinomas and sarcomas [17]. Malignant granular cell tumors tend to be larger, more rapidly growing, and located predominantly in the extremities when compared to the smaller, benign granular cell tumors seen in the head and neck regions. Most cases reported in old literature as malignant granular cell myoblastomas were later reviewed and were found to be alveolar sarcomas and other malignancies [6].

Granular cells are not unique to granular cell tumors as cytoplasmic granularity typical of GCT (both benign and malignant) has been observed in neoplastic and in non-neoplastic conditions, such as ameliobiastoma [17], amelioblastic fibroma [18,19], severed nerves undergoing Wallerian degeneration [20], traumatized muscle [21], leiomyosarcoma [22], angiosarcoma and appendiceal granular cell lesions.

GCT is rarely diagnosed prior to microscopic examination of the biopsy or excised specimen and often is an incidental finding during routine physical examination. As a rule, GCTs generally tend to follow a benign clinical course and of the nine previously reported cases of GCT of the urinary bladder, only one was malignant (Table 1). Conservative surgical treatment such as transurethral resection alone or partial cystectomy appears to offer adequate means of local control for benign tumors and more radical resections are not required. It is crucial that these benign tumors be clearly differentiated from much more common malignant solid tumors of the urinary bladder to save the patient from radical management protocols. In our first case, for instance, conservative surgical treatment was adequate. Given the limited information, it appears appropriate to recommended periodic follow up of these cases to rule out recurrence.

Malignant GCT although well established is extremely rare and poses a difficult diagnostic problem. Benign and malignant GCT could be similar in histological appearance. Clinical features such as rapid growth, presence of metastases and local recurrence generally indicates the tumor to be malignant. Microscopic features that favor a diagnosis of malignancy include cellular growth in sheets and clusters with invasion, nuclear hyperchromasia, presence of necrosis, and pleomorphism. Malignant GCTs tend to be slightly more cellular with smaller cells assuming spindle cell morphology. It has been noted that cellular variability or pleomorphism alone is not always a reliable diagnostic criterion. Other features especially when seen in combination such as necrosis, large vesicular nuclei with large nucleoli and high MIB-1 values favor malignancy. There is also an appreciable mitotic rate, although not apparent microscopically, 2 or more mitotic figures10/HPF should raise the suspicion of malignancy [14,17].

Our second case had features and histologic evidence of malignancy. We therefore opted for radical cystectomy. The patient remains free of disease recurrence 8 years since surgery and we recommend radical cystectomy as the standard therapy for invasive malignant GCT with no metastasis.

## Conclusion

Granular cell tumors (GCTs) are extremely rare lesions of the urinary bladder. They mostly follow a clinically benign course but are commonly mistaken for malignant tumors since they are solid looking, ulcerated tumors with ill-defined margins. We recommend careful pathologic assessment for establishing the appropriate diagnosis and either a conservative or aggressive surgical treatment for benign or localized malignant GCT of the urinary bladder, respectively.

## Conflict of interests

The author(s) declare that they have no competing interests.

## Authors' contributions

FA: Primary author and consultant urologist

AM: Contributing author and consultant urologist

TS: Research officer and performed literature review

NK: Consultant pathologist who performed pathological assessment of the case, contributing author

NAA: Fellow Urology, involved in data collection, pathological assessment and report preparation

All authors read and approved final version of the manuscript for publication

## References

[B1] Ravich A, Stout AP, Ravich RA (1945). Malignant granular cell myoblastoma involving the urinary bladder. Ann Surg.

[B2] Andersen R, Hoeg K (1961). Myoblastoma of the bladder neck: report of a case. Br J Urol.

[B3] Seery WH (1968). Granular cell myoblastoma of the bladder: report of a case. J Urol.

[B4] Okuda N, Okawa T, Nakamura T, Ishida O, Uchida H (1969). Granular cell myoblastoma of the urinary bladder: report of a case. Hinyokika Kiyo.

[B5] Christ ML, Ozzello L (1971). Myogenous origin of a granular cell tumor of the urinary bladder. Am J Clin Pathol.

[B6] Mouradian JA, Coleman JW, McGovern JH, Gray GF (1974). Granular cell tumor (Myoblastoma) of the bladder. J Urol.

[B7] Fletcher MS, Aker M, Hill JT, Pryor JP, Whimster WF (1985). Granular cell myoblastoma of the bladder. Br J Urol.

[B8] Kontani K, Okaneya T, Takezaki T (1999). Recurrent granular cell tumor of the bladder in a patient with von Recklinghausen's disease. BJU International.

[B9] Yoshida T, Hirai S, Horii Y, Yamauchi T (2001). Granular cell tumor of the urinary bladder: case report. Int J Urol.

[B10] Abrikossof A (1926). Ueber Myome ausgehened von der quergestreiften willkuerlichen Muskulatur. Virchows Arch (Patho Anat).

[B11] Sobel HJ, Schwarz R, Marquet R (1973). Light and electron microscope study of the origin of granular cell myoblastoma. J Pathol.

[B12] LeBoit PE, Barr RJ, Burall S, Metcalf JS, Yen TS, Wick MR (1991). Primitive polypoid granular cell tumor and other cutaneous granular cell neoplasms of apparent non neural origin. Am J Surg Pathol.

[B13] Victoria LV, Hoffman HT, Robinson RA (1998). Granular cell tumor of the larynx. J Laryngol Otol.

[B14] Rosai (2004). Soft tissues chapter. Ackerman's, Surgical pathology.

[B15] Miettinen M, Lehtonen E, Lehtola H, Ekblom P, Lehto VP, Virtanen I (1984). Histogenesis of granular cell tumor: an immunological and ultrastructural study. J Pathol.

[B16] Mukai M (1983). Immunohistochemical localization of S-100 protein and peripheral nerve myelin proteins (P2 protein and P0 protein) in granular cell tumors. Am J Pathol.

[B17] (2001). Benign tumors of peripheral nerves chapter. Enzinger and Weiss's Soft Tissue Tumors.

[B18] Nakazato Y, Ishizeki J, Takahashi K, Yamagushi H (1982). Immunohistochemical localization of S-100 Protein in granular cell myoblastoma. Cancer.

[B19] Navarrette AR, Smith M (1971). Ultrastructure of granular cell ameloblastoma. Cancer.

[B20] Couch DR, Morris EE, Velliou F (1952). Granular cell ameloblastic fibroma: report of two cases in adult, with observations on its similarity to congenital epilu. Am J Clin Pathol.

[B21] Waldron CA, Thompson CW, Conner WA (1963). Granular cell ameloblastic fibroma: report of two cases. Oral Surg Oral Med Oral Pathol.

[B22] Fisher ER, Wechsler H (1962). Granular cell myoblastoma – a misnomer: electron microscopic and histochemical evidence concerning its Schwann cell derivation and nature (granular cell schwannoma). Cancer.

[B23] Sobel JH, Marquet E (1974). Granular cells and granular cell lesions. Patho Annu.

[B24] Nistal M, Paniagua R, Picazo ML, Cermeno de Giles F, Ramos Guerreira JL (1980). Granular changes in vascular leiomyosarcoma. Virchows Arch A Pathol Anat Histol.

